# Mental health monitoring in adolescents with SLE: associations with lupus low disease activity state and remission

**DOI:** 10.1136/lupus-2026-002034

**Published:** 2026-06-17

**Authors:** Chayanit Kongsuk, Navaporn Tangkittiwet, Sirirat Charuvanij, Yanarin Thunsiribuddhichai, Anirut Pattaragarn, Nuntawan Piyaphanee

**Affiliations:** 1 Department of Pediatrics, Faculty of Medicine Siriraj Hospital, Mahidol University, Bangkok, Thailand

**Keywords:** Lupus Erythematosus, Systemic, Patient Reported Outcome Measures, Health-Related Quality Of Life, Outcome Assessment, Health Care

## Abstract

**Objective:**

Psychological comorbidities are common in adolescents with SLE. Incorporating structured mental-health screening into adolescent SLE care may facilitate improved disease control and holistic well-being. We aimed to evaluate evidence describing changes in mental health including psychological comorbidities and associations with disease states in adolescents with SLE.

**Methods:**

A prospective cohort study was conducted in adolescents aged 12–18 years diagnosed with SLE and completed paired assessments at two visits 6–12 months apart. Mental-health measures included the Patient Health Questionnaire-Adolescents (PHQ-A) (depressive symptoms), Generalised Anxiety Disorder (GAD-7) (anxiety), PedsQL Multidimensional Fatigue Scale (PedsQL-MFS) (fatigue), Pittsburgh Sleep Quality Index (PSQI) (sleep quality), Brief Illness Perception Questionnaire (B-IPQ) (illness perception) and visual analogue scale for pain (VAS-P) (pain). Disease states comprised SLE Disease Activity Index 2000 (SLEDAI-2K) (disease activity) and attainment of childhood lupus low disease activity state (cLLDAS) or childhood-onset SLE (cSLE) remission. Group comparisons, paired analyses and correlation analyses were performed.

**Results:**

120 participants were enrolled, of which 117 had complete paired assessments. Most participants were females (90.5%), with a mean age of 15.1±0.2 years. Significant improvement in mental health measures was observed including PHQ-A (3 (1, 6) vs 2 (1, 5), p=0.013), GAD-7 (2 (1, 5) vs 2 (0, 4), p=0.004) and PSQI (4.6±2.8 vs 3.9±2.4, p=0.015) scores, whereas PedsQL-MFS, B-IPQ and VAS-P scores remained unchanged. SLEDAI-2K scores were low (2 (0, 4) vs 2 (0, 3)). The proportions of achieving cLLDAS (64.1% vs 72.6%, p=0.122) and cSLE remission (27.4% vs 35.9%, p=0.021) increased. The relationship between the psychological measures and SLEDAI-2K was not observed; however, active disease (SLEDAI-2K ≥5) was associated with greater fatigue (p=0.015) at the second visit. No associations were identified between the psychological measures and cLLDAS or cSLE remission, consistently in both visits.

**Conclusions:**

Mental health monitoring over 6–12 months apart revealed changes in depressive symptoms, anxiety and sleep quality during routine paediatric SLE care. The absence of associations with cLLDAS or remission underscores the importance of integrating mental health outcomes into the treat-to-target strategies.

**WHAT IS ALREADY KNOWN ON THIS TOPIC**

- Adolescents with SLE frequently experience depression, anxiety, fatigue and sleep disturbances, but the longitudinal relationship between mental health status and disease states remains poorly defined.

**WHAT THIS STUDY ADDS**

- This study shows that depressive symptoms, anxiety and sleep quality improved over 6–12 months in adolescents with SLE, but these psychological changes were not associated with childhood lupus low disease activity state or childhood-onset SLE remission status.
- Fatigue remained unchanged and was inconsistently associated with active disease (SLE Disease Activity Index 2000 ≥5), supporting its persistent and multifactorial nature in adolescent SLE.

**HOW THIS STUDY MIGHT AFFECT RESEARCH, PRACTICE OR POLICY**

- Integrating patient-reported mental-health measures into paediatric SLE care may support a more holistic approach alongside disease activity–focused management.
- Larger longitudinal studies are needed to clarify how psychological outcomes can be incorporated into future treat-to-target strategies in SLE.

## Introduction

 SLE, a chronic, multisystem autoimmune disease that frequently presents during adolescence, is characterised by a relapsing–remitting course and substantial treatment burden.^1 2^ In addition to organ-specific damages, adolescents with SLE experience a high prevalence of psychological comorbidities, including depressive symptoms, anxiety, fatigue, sleep disturbance and maladaptive illness perceptions.^3^,^6^ These mental health challenges may be recognised as important determinants of health-related quality of life, treatment adherence and long-term outcomes in paediatric SLE.^4 7 8^ Mental health screening is recommended for all adolescents annually according to the American Academy of Pediatrics^9^ and is increasingly demonstrated an acceptability, feasibility and effectiveness to identify mental health problems in paediatric SLE.^10 11^

The management of SLE has shifted toward a treat-to-target (T2T) strategy, which emphasises regular assessment and timely adjustment of therapy to achieve predefined disease activity targets—preferably remission or, alternatively, lupus low disease activity state (LLDAS).^12^,^15^ Achievement and maintenance of these targets are associated with reduced disease flares, lower damage accrual and improved survival.^16^,^20^ However, T2T frameworks have traditionally focused on physician-reported clinical measures, with limited integration of patient-reported outcomes, particularly mental-health indicators. In adolescents, who are navigating critical developmental, educational and psychosocial transitions, mental health burden may not parallel improvements in disease activity and may remain under-recognised in routine care.

Despite growing awareness of psychological morbidity in paediatric SLE, longitudinal evidence describing changes in mental health over time and their relationship with disease states including disease activity, LLDAS and remission remain scarce.^11 12 21^ Most existing studies are cross-sectional or focus on single psychological domains, limiting the understanding of how mental-health trajectories evolve during routine follow-up and whether they align with attainment of LLDAS or remission. Incorporating structured mental-health screening into paediatric SLE care may facilitate more comprehensive disease management and improved overall well-being.

Recently, the childhood LLDAS (cLLDAS) and the childhood-onset SLE (cSLE) remission have been defined and applied in cSLE cohorts.^22^,^24^ Therefore, the objective of this study was to evaluate changes in psychological outcomes over 6–12 months among adolescents with SLE and to examine their associations with disease activity, achievement of cLLDAS and cSLE remission. By characterising longitudinal mental-health trajectories alongside clinical disease states, this study aims to inform the role of routine psychological monitoring in optimising T2T-based care for adolescents with SLE.

## Methods

### Study design and participants

This prospective cohort study was performed following a prior cross-sectional analysis of physical and mental health in adolescents with SLE.^6^ This study was conducted in paediatric-onset SLE adolescents aged 12–18 years follow-up at Rheumatology Clinic and Nephrology Clinic at Siriraj Hospital, Mahidol University, Bangkok, Thailand, between October 2022 and April 2025. SLE was classified based on the 1997 American College of Rheumatology (ACR) criteria, the 2012 Systemic Lupus International Collaborating Clinics criteria or the 2019 European Alliance of Associations for Rheumatology (EULAR/ACR) classification criteria.^25^,^27^ Participants who were able to understand and independently complete two-time point self-administered questionnaires during 6–12 months period were included, while those who had a documented diagnosis of major depressive disorder before SLE onset or were pregnant during the study period were excluded.

### Measurement by questionnaires

Participants completed a Thai-language, paper-based questionnaire comprising demographic information, psychosocial variables and validated psychological assessment tools at two time points, 6–12 months apart. Psychosocial information and patient’s perspectives included parental marital status, family income sufficiency, relationships with family members and peers, and current schooling or employment status. Depressive symptoms were assessed with the Patient Health Questionnaire-Adolescents (PHQ-A), a 9-item tool validated among Thai adolescents; scores equal to and greater than 5 were considered indicative of depressive symptoms.^28^ Anxiety symptoms were assessed using the 7-item Generalised Anxiety Disorder Scale (GAD-7).^29^ Sleep quality was evaluated using the Pittsburgh Sleep Quality Index (PSQI), with scores equal to and more than 5 representing poor sleep quality.^30 31^ Fatigue was measured using the PedsQL Multidimensional Fatigue Scale (PedsQL-MFS), which includes general, sleep/rest and cognitive fatigue subscales; scores were reverse-scored and transformed to a 0–100 scale, where higher scores indicate fewer fatigue symptoms.^32^ Illness perception was evaluated using the 9-item Brief Illness Perception Questionnaire (B-IPQ), which measures cognitive and emotional representations of illness across eight domains using 0–10 numeric scales; items 3, 4 and 7 were reverse-scored, and higher total scores indicated more negative illness perception.^33^ Pain severity was assessed using a 10 cm visual analogue scale (VAS-P), in which values greater than 3 indicated clinically significant pain. Suicidal ideation was recorded as part of the PHQ-A instrument. All psychological assessment instruments were administered using Thai-language versions. The PHQ-A has been formally validated in Thai adolescent populations, while the remaining instruments have validated Thai translations with established psychometric properties.^6 31 34^

### Clinical and outcome assessments

Each study visit, laboratory evaluations included complete blood count, erythrocyte sedimentation rate (ESR), serum creatinine, urine protein–creatinine ratio (UPCR), complement C3 and C4, and anti-double-stranded DNA antibodies. Current clinical features of SLE and treatment were collected. SLE Disease Activity Index 2000 (SLEDAI-2K) and the Physician Global Assessment (PGA; 0–3 scale) were assessed.^35^ ^36^ Medical records were reviewed for age at SLE onset, psychological illness after diagnosed SLE, history of neuropsychiatric SLE and history of biopsy-proven proliferative lupus nephritis (LN).

Disease states of SLE were determined by achieving the cLLDAS and cSLE remission.^22 23^ cLLDAS was defined according to international consensus criteria: a SLEDAI-2K score ≤4 with no activity in major organ systems (renal, central nervous system, cardiopulmonary, vasculitis or fever), absence of haemolytic anaemia or gastrointestinal activity, no new disease activity compared with the previous assessment, a PGA score ≤1, a daily prednisolone dose of ≤0.15 mg/kg/day (maximum 7.5 mg/day) and use of stable antimalarials, well-tolerated maintenance immunosuppressive or biological therapy.^22^ The assessment of cSLE remission was based on the Paediatric Rheumatology European Society endorsed consensus definitions. cSLE remission was defined as meeting all of the following components: clinical SLEDAI-2K=0; a PGA score <0.5; stable antimalarials, immunosuppressive and biological agents; and prednisolone ≤0.10 mg/kg/day (maximum 5 mg/day).^23^ Additionally, SLEDAI score ≥5 was defined as having active SLE.

### Statistical analysis

Descriptive statistics were used to summarise demographic and clinical characteristics, presented as mean±SD for normally distributed continuous variables, median with IQRs 25th, 75th for non-normally distributed variables and frequencies with percentages for categorical variables. Comparisons between first and second visits were conducted using paired t-tests for normally distributed continuous variables, Wilcoxon signed-rank tests for non-normally distributed continuous variables and McNemar’s test for paired categorical variables. Between-group comparisons at each follow-up (eg, active SLE vs non-active SLE, cLLDAS vs non-cLLDAS, cSLE remission vs non-remission and history of LN vs no history) were performed using independent t-tests or Mann–Whitney U tests for continuous variables, and χ² for categorical variables. Correlations between psychological scores, sleep quality, fatigue, illness perception and disease activity indices were assessed using Pearson’s correlation coefficient for normally distributed variables and Spearman’s rank correlation for non-normally distributed variables. A correlation coefficient (r) ranges from −1 to 1. Values from 0.6 to 1.0 (or −0.6 to 1.0) indicate a strong positive (or negative) correlation, 0.4 to 0.59 (or −0.4 to −0.59) represents a moderate correlation, 0.2 to 0.39 (or −0.2 to −0.39) reflect a weak correlation and less than 0.2 (or between −0.19 and 0) indicate no correlation. All analyses were performed using IBM SPSS Statistics V.29.0 (IBM, Armonk, New York, USA). Two-sided p values <0.05 were considered statistically significant.

### Patient and public involvement

Patients and the public were not directly involved in the design, conduct, reporting or dissemination plans of our research on this study or in the analysis of data.

## Results

### Baseline sociodemographic and clinical characteristics

A total of 120 adolescents with SLE were enrolled at the first visit. At the second study visit, 3 participants did not complete the questionnaires; therefore, 117 paired observations were eligible for longitudinal analysis. There were no patients with major depression before SLE diagnosis or pregnant during the study period. Baseline characteristics at the study entry are shown in table 1. The majority of the participants were females (90.5%), with a mean age of 15.1±0.2 years. Self-reported personal data revealed divorced/separated parental-marital status of 37.6%, insufficient family incomes 21.3%, having poor relationship with family members 23.9%, drop out of school or unemployed 5.1% and having poor relationship with friends 15.4%. 15.4% of the participants developed psychological illnesses (including depressive, anxiety, adjustment and learning disorders) after diagnosing SLE. 11.9% of the patients had history of neuropsychiatric SLE and 64.1% experienced proliferative LN. Most participants received hydroxychloroquine (94%) and prednisolone (94.8%), while 57.2% treated with immunosuppressive drugs (57.2%), mainly mycophenolate mofetil (28.2%) and azathioprine (28.2%). Over a mean follow-up period of 8.2±0.2 months, the prevalence of organ manifestations—including mucocutaneous, musculoskeletal, haematological and renal involvement—remained comparable between visits. Laboratory results between the first and the second visit included haemoglobin, white blood cell count, platelet count, ESR, UPCR, C3 and C4 levels were not significant differences, while serum creatinine at the follow-up visit was slightly increased (0.6±0.2 vs 0.6±0.3 mg/dL, p=0.025) (online supplemental table 1).

**Table 1:** Baseline characteristics of adolescents with SLE at the study entry

Characteristics	Total (n=117)
Female, n (%)	106 (90.5)
Age at the first visit, years; mean±SD	15.1±0.2
Age at diagnosis, years; mean±SD	11.5±0.2
Divorced/separated parental-marital status*, n (%)	44 (37.6)
Insufficient family incomes*, n (%)	25 (21.3)
Poor relationship with family members*, n (%)	28 (23.9)
Drop out of school/unemployed*, n (%)	6 (5.1)
Poor relationship with friends*, n (%)	18 (15.4)
Ever psychological illness after diagnosed SLE, n (%)	18 (15.4)
Depression	9 (7.6)
Anxiety	2 (1.7)
Adjustment disorder	7 (6.0)
Learning disorder	6 (5.1)
Neuropsychiatric SLE, n (%)	14 (11.9)
Proliferative lupus nephritis, n (%)	75 (64.1)
Haemoglobin, g/L; mean±SD	11.8±1.6
White blood cell count, x10^9^/L; mean±SD	6.69±2.46
Platelet count, x10^9^/L; mean±SD	300.15±79.79
Erythrocyte sedimentation rate, mm/hour; mean±SD	16.5 (8, 32.8)
Serum creatinine, mg/dL; mean±SD	0.6±0.2
Urine protein per creatinine ratio, mg/mg; median (IQR)	0.1 (0.1, 0.2)
Complement C3 level, mg/dL; mean±SD (n=114)	92.7±25.6
Complement C4 level, mg/dL; mean±SD (n=74)	17.7±9.1
Presence of anti-dsDNA, n (%) (n=104)	49 (47.1)
Treatment, n (%)	
Hydroxychloroquine	110 (94.0)
Prednisolone	111 (94.8)
Immunosuppressive agents	67 (57.2)
Mycophenolate mofetil	33 (28.2)
Azathioprine	33 (28.2)
Calcineurin inhibitor	4 (3.4)
Intravenous cyclophosphamide	2 (1.7)
Methotrexate	1 (0.8)

* Self-reported personal data

anti-dsDNA, anti-double-stranded DNA.

### SLE disease activity, disease states and mental health measurement at follow-up visits 6–12 months apart

SLE disease activity, disease states and mental health measures at the first and second visits are summarised in table 2. Disease activity, assessed by the SLEDAI-2K, showed a statistically significant reduction (p=0.021); however, the median score remained unchanged at two with overlapping IQR (0–4 vs 0–3), indicating persistently low disease activity at both visits. Compared with the first visit, the proportion of patients achieving cLLDAS increased from 64.1% to 72.6%, although this did not reach statistical significance (p=0.122), while the proportion achieving cSLE remission increased significantly from 27.4% to 35.9% (p=0.021). In contrast, the proportion of patients with cSLE remission off steroid, active SLE (SLEDAI-2K ≥5), PGA scores and prednisolone doses did not differ significantly between visits.

**Table 2:** Comparisons of mental health measurement and SLE disease states between the first and the second visits

Parameters	First visit (n=117)	Second visit (n=117)	P value
SLEDAI-2K, score; median (IQR)	2 (0, 4)	2 (0, 3)	0.021*
Active SLE (SLEDAI-2K score≥5), n (%)	18 (15.4)	14 (12.0)	0.503
PGA (scale 0–3), score; median (IQR)	0 (0, 0.3)	0 (0, 0.3)	0.064
Current prednisolone, mg/day; median (IQR)	5 (5, 10)	5 (5, 5)	0.493
Current prednisolone, mg/kg/day; median (IQR)	0.12 (0.08, 0.17)	0.10 (0.07, 0.13)	0.277
cLLDAS, n (%)	75 (64.1)	85 (72.6)	0.122
cSLE remission, n (%)	32 (27.4)	42 (35.9)	0.021*
cSLE remission with no steroid use, n (%)	3 (2.6)	5 (4.3)	0.500
PHQ-A, score; median (IQR)	3 (1, 6)	2 (1, 5)	0.013*
GAD-7, score; median (IQR)	2 (1, 5)	2 (0, 4)	0.004*
PSQI, score; mean±SD	4.6±2.8	3.9±2.4	0.015*
PedsQL-MFS, total score; mean±SD	76.6±15.8	77.3±16.0	0.563
General	81.1±16.6	82.8±15.1	0.266
Sleep/rest	72.9±19.9	72.6±20.0	0.832
Cognitive	76.3±8.8	76.7±20.1	0.829
B-IPQ, score; median (IQR)	20 (11, 30)	18 (11, 29)	0.394
Consequence	1 (0, 3.5)	1 (0, 4)	0.912
Timeline	5 (2, 8)	4 (1, 8)	0.515
Personal control (reversed score)	2 (0, 5)	2 (0, 5)	0.116
Treatment control (reversed score)	0 (0, 2)	0 (0, 2)	0.894
Identity	1 (0, 3)	1 (0, 3)	0.262
Concern	1 (0, 4)	1 (0, 4)	0.899
Understanding (reversed score)	1 (0, 2.5)	1 (0, 2)	0.123
Emotional response	3 (0, 5)	2 (0, 5)	0.132
VAS-P, score; median (IQR)	0 (0, 1.5)	0 (0, 2)	0.416

*p<0.05 is considered a significant level.

B-IPQ, Brief Illness Perception Questionnaire; cLLDAS, childhood lupus low disease activity state; cSLE, childhood-onset SLE; GAD-7, Generalised Anxiety Disorder-7; PedsQL-MSF, PedsQL Multidimension Fatigue Scale; PGA, Physician Global Assessment; PHQ-A, Patient Health Questionnaire for Adolescents; PSQI, Pittsburgh Sleep Quality Index; SLEDAI-2K, SLE Disease Activity Index 2000; VAS-P, visual analogue scale for pain.

During mental-health monitoring conducted 6–12 months apart, significant changes were observed in depressive symptoms, anxiety and sleep quality. PHQ-A scores decreased from 3 (1, 6) to 2 (1, 5) (p=0.013), GAD-7 scores declined from 2 (1, 5) to 2 (0, 4) (p=0.004) and PSQI scores improved from 4.6±2.8 to 3.9±2.4 (p=0.015). In contrast, PedsQL-MFS for fatigue (76.6±15.8 vs 77.3±16.0, p=0.563), B-IPQ for illness perception (20 (11, 30) vs 18 (11, 29), p=0.394) and VAS-P scores for pain (0 (0, 1.5) vs 0 (0, 2), p=0.416) did not differ significantly between the two visits.

### Correlation analysis of mental health measures and disease activity

The PHQ-A (r=0.021, p=0.615), GAD-7 (r=0.035, p=0.822), PSQI (r=0.021, p=0.822), PedsQL-MFS (r=−0.076, p=0.481), B-IPQ (r=0.025, p=0.787) and VAS-P (r=−0.42, p=0.651) were not correlated with SLEDAI-2K, while SLEDAI-2K was moderately related to PGA (r=0.52, p<0.001). Correlation analysis among psychological measures was performed and observed strong to moderate intercorrelations among depression, anxiety, sleep disturbance, fatigue and illness perception (r=0.4–0.8). PHQ-A for depressive symptoms was strongly positively correlated to GAD-7 for anxiety and PSQI for sleep quality, while strongly negatively related to PedsQL-MFS which had fewer fatigue symptoms. PedsQL-MSF was also strongly negatively correlated with B-IPQ for illness perception. Moderate interrelations were observed among GAD-7, PSQI and B-IPQ. VAS-P had weak to moderate correlations with PHQ-A, GAD-7, PSQI, B-IPQ and PedsQL-MFS. Additionally, prednisolone dose did not correlate to any psychological measures (online supplemental table 2).

### Association of mental health measures and SLE disease states

Each mental health measurement was compared between achieved cLLDAS and non-achieved cLLDAS at both visits. PHQ-A, GAD-7, PSQI, PedsQL-MFS, B-IPQ and VAS-P scores were not associated with cLLDAS (table 3). No associations were identified between these psychological measures and cSLE remission (table 4). At the second visit, lower total score of PedsQL-MFS (greater fatigue) was observed in patients with active SLE (SLEDAI-2K score ≥5) compared with those inactive SLE (SLEDAI-2K <5) (table 5). Additionally, patients who had history of LN had significantly higher SLEDAI-2K (p=0.025), PGA (p=0.022) and steroid requirements (p=0.021) at second visits, but psychological profiles did not differ significantly from those without LN (online supplemental table 3).

**Table 3:** Mental health status between achieved cLLDAS and non-achieved cLLDAS in adolescents with SLE

Mental health parameters	First visit		P value	Second visit		P value
	Achieved cLLDAS (n=75)	Non-achieved cLLDAS (n=42)		Achieved cLLDAS (n=85)	Non-achieved cLLDAS (n=32)	
PHQ-A, score; median (IQR)	3 (1.5, 5)	3 (1, 6)	0.825	2.5 (1, 5)	2 (1, 5)	0.946
GAD-7, score; median (IQR)	3 (0, 5)	2 (1, 5)	0.759	1.5 (0, 3)	2 (0, 4.5)	0.321
PSQI, score; median (IQR)	3 (2, 6)	4 (3, 6.75)	0.169	4 (2, 6)	4 (2, 6)	0.966
PedsQL-MFS, total score; mean±SD	76.7±12.6	76.5±17.4	0.704	75.9±16.6	77.8±15.8	0.568
General	81.8±12.5	80.8±12.2	0.845	81.4±14.2	83.4±15.5	0.522
Sleep/rest	72.0±16.9	73.4±21.4	0.582	69.4±23.2	73.8±18.7	0.292
Cognitive	77.5±15.5	75.7±20.5	0.823	77.2±21.1	76.5±19.8	0.869
B-IPQ, score; median (IQR)	18 (9, 34)	20.5 (11, 30)	0.873	18 (10.5, 31)	17 (11, 28.5)	0.957
VAS-P, score; median (IQR)	0 (0, 1)	0 (0, 2)	0.853	0 (0, 1.75)	0 (0, 3)	0.817

p<0.05 is considered a significant level.

B-IPQ, Brief Illness Perception Questionnaire; cLLDAS, childhood lupus low disease activity state; GAD-7, Generalised Anxiety Disorder-7; PedsQL-MSF, PedsQL Multidimension Fatigue Scale; PHQ-A, Patient Health Questionnaire for Adolescents; PSQI, Pittsburgh Sleep Quality Index; VAS-P, visual analogue scale for pain.

**Table 4:** Mental health status between attained cSLE remission and non-attained cSLE remission in adolescents with SLE

Mental health parameters	First visit		P value	Second visit		P value
	cSLE-remission (n=32)	Non-remission (n=85)		cSLE-remission (n=42)	Non-remission (n=75)	
PHQ-A, score; median (IQR)	3 (1, 6)	3 (1, 6)	0.202	2 (1, 6)	2 (1, 5)	0.286
GAD-7, score; median (IQR)	2.5 (1, 7.25)	2 (1, 5)	0.242	1 (0, 5)	2 (1, 4)	0.499
PSQI, score; median (IQR)	4.5 (2, 8)	4 (2, 6)	0.269	3 (2, 6)	4 (2, 5)	0.961
PedsQL-MFS, total score; mean±SD	76.4±20.1	76.7±14.1	0.946	75.6±18.1	78.3±14.7	0.381
General	81.2±19.5	81.1±15.5	0.971	81.4±17.6	83.7±13.6	0.442
Sleep/rest	73.0±24.2	72.9±18.2	0.977	72.3±20.5	72.8±20.3	0.898
Cognitive	75.1±23.8	76.8±16.8	0.670	73.3±22.0	78.6±18.8	0.168
B-IPQ, score; median (IQR)	20.5 (11.6, 30.0)	19.0 (9.5, 31.5)	0.948	19.5 (10.5, 30.5)	17.0 (11.0, 26.0)	0.354
VAS-P, score; median (IQR)	0 (0, 1.8)	0 (0, 1.5)	0.898	0 (0, 3)	0 (0, 2)	0.211

p<0.05 is considered a significant level.

B-IPQ, Brief Illness Perception Questionnaire; cSLE, childhood-onset SLE; GAD-7, Generalised Anxiety Disorder-7; PedsQL-MSF, PedsQL Multidimension Fatigue Scale; PHQ-A, Patient Health Questionnaire for Adolescents; PSQI, Pittsburgh Sleep Quality Index; VAS-P, visual analogue scale for pain.

**Table 5:** Mental health outcomes between active SLE (SLEDAI score ≥5) and inactive SLE (SLEDAI <5) in adolescents with SLE

Variable	First visit		P value	Second visit		P value
	Active SLE (n=18)	Inactive SLE (n=99)		Active SLE (n=14)	Inactive SLE (n=103)	
PHQ-A, score; median (IQR)	2.5 (1, 6)	3 (1, 6)	0.761	4 (1.75, 6.75)	2 (1, 5)	0.114
GAD-7, score; median (IQR)	4 (0.75, 4)	2 (1, 5)	0.991	2 (1, 5)	2 (0, 4)	0.361
PSQI, score; (IQR)	3.5 (2, 6)	4 (3, 6)	0.306	4 (1.75, 8)	4 (2, 6)	0.527
PedsQL-MFS, total score; mean±SD	75.4±14.1	76.8±16.2	0.726	67.7±20.0	78.6±15.0	0.015*
General	80.3±15.2	81.3±17.0	0.826	74.7±16.6	84.0±14.6	0.030*
Sleep/rest	68.5±17.3	73.7±20.3	0.309	58.0±27.4	74.6±18.1	0.003*
Cognitive	77.3±16.4	76.2±19.3	0.812	70.5±26.3	77.6±19.1	0.220
B-IPQ, score; median (IQR)	23 (8.75, 34.5)	19 (11, 30)	0.535	21 (9.5, 36.25)	17 (11, 28)	0.275
VAS-P, score; median (IQR)	0 (0, 5)	0 (0, 1)	0.097	0 (0, 3)	0 (0, 2)	0.632

*p<0.05 is considered a significant level.

B-IPQ, Brief Illness Perception Questionnaire; GAD-7, Generalised Anxiety Disorder-7; PedsQL-MSF, PedsQL Multidimension Fatigue Scale; PHQ-A, Patient Health Questionnaire for Adolescents; PSQI, Pittsburgh Sleep Quality Index; SLEDAI-2K, SLE Disease Activity Index 2000; VAS-P, visual analogue scale for pain.

## Discussion

In this prospective study of adolescents with SLE, we observed modest but statistically significant improvements in depressive symptoms, anxiety and sleep quality over a 6–12 month follow-up period during routine clinical care. In contrast, fatigue, illness perception and pain remained largely unchanged. Although SLEDAI-2K scores decreased statistically, the median values remained low and unchanged, indicating minimal clinical change and persistently low disease activity at both visits. While the proportion of patients achieving cSLE remission increased, psychological measures were not associated with SLEDAI-2K, achievement of cLLDAS or remission status. These findings suggest that psychological outcomes may evolve independently of clinical disease activity, highlighting the complex and multidimensional nature of disease burden in adolescent SLE.

The observed improvements in depressive symptoms, anxiety and sleep quality may reflect resolution of disease activity, treatment optimisation and regular clinical engagement during follow-up. Adolescents with SLE are known to have high rates of depression, anxiety and sleep quality, and even small improvements may be clinically meaningful in this population.^3^,^7^ In contrast, fatigue did not improve, supporting prior evidence that fatigue is among the most persistent and treatment-resistant symptoms in SLE and is influenced by inflammatory activity and non-inflammatory factors such as physical deconditioning and psychosocial stress.^4 7 37 38^ Similarly, illness perception remained stable, suggesting that cognitive representations of illness may evolve independently of clinical status and reflect ongoing adjustment to living with a chronic condition; a longer duration of follow-up may be required to detect meaningful change. In addition, consistently low pain scores may indicate the absence of clinically significant pain in this cohort.

From a clinical perspective, disease activity remained persistently low at both visits, accompanied by an increase in the proportion of patients achieving SLE remission. The proportion achieving LLDAS also increased, although this did not reach statistical significance. These findings align with prior study demonstrating that sustained low disease activity or remission is achievable in a substantial proportion of paediatric patients under contemporary management strategies.^39^ However, despite these favourable clinical trends, psychological outcomes were not associated with SLEDAI-2K, LLDAS or remission status at either visit, highlighting a disconnect between physician-assessed disease activity and patient-reported mental health. T2T strategies in SLE primarily emphasise remission or LLDAS as therapeutic goals because of their associations with reduced flares and damage accrual.^1213 16^,^20^ However, our results suggest that achieving these clinical targets alone may not be sufficient to address holistic well-being in adolescents. Therefore, mental health should be considered in-keeping with T2T in paediatric SLE.^13^

The absence of correlations between most psychological measures and SLEDAI-2K is consistent with previous paediatric studies reporting discordance between disease activity indices and patient-reported outcomes.^4 6 7 10^ Depression, anxiety, fatigue and sleep disturbance often persist independently of disease status, suggesting that conventional disease activity measures may incompletely capture the real-life of adolescents living with SLE. Notably, active disease (SLEDAI-2K ≥5) was associated with greater fatigue at the second visit, supporting the concept that fatigue may be partially—but not exclusively—related to disease activity. This nuanced relationship reinforces fatigue as a key symptom bridging biological and psychosocial domains. Notably, this cohort was characterised by persistently low SLEDAI-2K scores, and failure to achieve cLLDAS or remission in some patients may have been influenced in part by ongoing prednisolone dose rather than SLE disease activity. Corticosteroids are known to affect mood, sleep and fatigue. However, in this cohort with low disease activity and generally low prednisolone doses, we did not observe any significant associations between prednisolone dose and psychological measures. These findings suggest that, within this low-disease-activity context, neither disease activity nor current corticosteroid exposure was a major determinant of mental health outcomes.

Importantly, strong to moderate intercorrelations were observed among depression, anxiety, sleep disturbance, fatigue and illness perception, highlighting the clustering of psychological and symptom-related domains in adolescent SLE. This pattern has been described previously^4 6 7^ and may suggest shared underlying mechanisms, including chronic inflammation, hypothalamic–pituitary–adrenal axis dysregulation and psychosocial stressors.^38^ ^40^ These interrelationships support the need for comprehensive psychological assessment rather than isolated screening of single symptoms.

Taken together, these results support the integration of structured mental-health screening into routine paediatric SLE care. The current T2T and management recommendations acknowledge the importance of health-related quality of life and patient-reported outcomes; they explicitly incorporate psychological targets into treatment algorithms.^41^ In paediatric populations, a more holistic approach that integrates mental-health monitoring alongside disease activity targets may better address the full burden of disease.^13^

Several limitations should be acknowledged. The relatively short follow-up period and modest sample size may have limited the ability to detect associations between psychological outcomes and disease states such as cLLDAS or remission. The generally low SLEDAI-2K scores suggest that this cohort may not fully represent the broader paediatric SLE population, particularly patients with higher disease activity. In addition, cumulative or duration-based corticosteroid exposure was not assessed and may have influenced the observed psychological outcomes. Mental health assessment relied on widely used self-reported questionnaires rather than formal psychiatric diagnoses. Furthermore, the absence of a healthy Thai adolescents control group limits comparison with normative trajectories and may have introduced cultural and age-related measurement bias. Nevertheless, the longitudinal design and concurrent assessment of disease activity and T2T-related outcomes strengthen the relevance of our findings to routine paediatric rheumatology practice.

In conclusion, adolescents with SLE demonstrated significant improvements in depressive symptoms, anxiety and sleep quality over 6–12 months of follow-up, alongside improvements in disease activity and remission rates. However, psychological outcomes were largely independent of disease severity, LLDAS and remission status, with the exception of an association between active disease and fatigue. These findings highlight the importance of systematic mental-health monitoring as a complementary component of T2T–oriented care in paediatric SLE and support further investigation in larger, longer-term cohorts.

## Supplementary material

10.1136/lupus-2026-002034 online supplemental table 1

## Data Availability

All data relevant to the study are included in the article or uploaded as supplementary information.

## References

[1] Kamphuis S, Silverman ED (2010). Prevalence and burden of pediatric-onset systemic lupus erythematosus. Nat Rev Rheumatol.

[2] Groot N, de Graeff N, Avcin T (2017). European evidence-based recommendations for diagnosis and treatment of childhood-onset systemic lupus erythematosus: the SHARE initiative. Ann Rheum Dis.

[3] Quilter MC, Hiraki LT, Korczak DJ (2019). Depressive and anxiety symptom prevalence in childhood-onset systemic lupus erythematosus: A systematic review. Lupus (Los Angel).

[4] Jones JT, Cunningham N, Kashikar-Zuck S (2016). Pain, Fatigue, and Psychological Impact on Health-Related Quality of Life in Childhood-Onset Lupus. Arthritis Care Res (Hoboken).

[5] Baba O, Kisaoglu H, Bilginer C (2022). Depression, anxiety, and sleep quality in childhood onset systemic lupus erythematosus and relationship with brain-derived neurotrophic factor. Lupus (Los Angel).

[6] Tangkittiwet N, Charuvanij S, Manaboriboon B (2025). Illness perception and psychological distress in adolescents with systemic lupus erythematosus. Lupus (Los Angel).

[7] Donnelly C, Cunningham N, Jones JT (2018). Fatigue and depression predict reduced health-related quality of life in childhood-onset lupus. Lupus (Los Angel).

[8] Putera AM, Irwanto I, Maramis MM (2020). Effect of Mental Health Problems on the Quality of Life in Children with Lupus Nephritis. Neuropsychiatr Dis Treat.

[9] Weitzman C, Guevara J, Curtin M (2025). AAP Section on Developmental and Behavioral Pediatrics; AAP Council on Early Childhood; AAP Committee on Psychosocial Aspects of Child and Family Health; SOCIETY FOR DEVELOPMENTAL AND BEHAVIORAL PEDIATRICS. Promoting Optimal Development: Screening for Mental Health, Emotional, and Behavioral Problems: Clinical Report. Pediatrics.

[10] Rubinstein TB, Dionizovik‐Dimanovski M, Davis AM (2023). Multicenter Study of Utility and Acceptability of Depression and Anxiety Screening in Adolescents and Young Adults With Childhood‐Onset Systemic Lupus. Arthritis Care &Amp; Research.

[11] El Tal T, Chen A, Wong S (2024). Improving routine mental health screening for depression and anxiety in a paediatric lupus clinic: a quality improvement initiative for enhanced mental healthcare. Lupus Sci Med.

[12] Piga M, Parodis I, Touma Z (2025). Framework for implementing treat-to-target in systemic lupus erythematosus routine clinical care: consensus statements from an international task force. Autoimmun Rev.

[13] Smith EMD, Aggarwal A, Ainsworth J (2023). Towards development of treat to target (T2T) in childhood-onset systemic lupus erythematosus: PReS-endorsed overarching principles and points-to-consider from an international task force. Ann Rheum Dis.

[14] Franklyn K, Lau CS, Navarra SV (2016). Definition and initial validation of a Lupus Low Disease Activity State (LLDAS). Ann Rheum Dis.

[15] van Vollenhoven RF, Bertsias G, Doria A (2021). 2021 DORIS definition of remission in SLE: final recommendations from an international task force. Lupus Sci Med.

[16] Na Nakorn K, Piyaphanee N, Sukharomana M (2023). Outcomes of achieving lupus low disease activity state and damage accrual in childhood-onset systemic lupus erythematosus. Clin Rheumatol.

[17] Golder V, Kandane-Rathnayake R, Li N (2024). Association of sustained lupus low disease activity state with improved outcomes in systemic lupus erythematosus: a multinational prospective cohort study. Lancet Rheumatol.

[18] Kandane-Rathnayake R, Golder V, Louthrenoo W (2025). Association of lupus low disease activity state and remission with reduced organ damage and flare in systemic lupus erythematosus patients with high disease activity: a multi-national, longitudinal cohort study. Rheumatology (Oxford).

[19] Sarker C, Jorgensen AL, Tharmaratnam K (2025). Validation of childhood lupus specific targets: ensuring accurate assessment of disease control in younger, lighter paediatric patients. Rheumatology (Oxford).

[20] Yin W, Lin Y, Wang S (2026). Treat-to-target achievement and predictive analysis in childhood-onset systemic lupus erythematosus. Rheumatology (Oxford).

[21] Parodis I, Girard-Guyonvarc’h C, Arnaud L (2024). EULAR recommendations for the non-pharmacological management of systemic lupus erythematosus and systemic sclerosis. Ann Rheum Dis.

[22] Smith EMD, Aggarwal A, Ainsworth J (2023). PReS-endorsed international childhood lupus T2T task force definition of childhood lupus low disease activity state (cLLDAS). Clin Immunol.

[23] Smith EMD, Aggarwal A, Ainsworth J (2024). Defining remission in childhood-onset lupus: PReS-endorsed consensus definitions by an international task force. Clin Immunol.

[24] Bergkamp SC, Kanagasabapathy T, Gruppen MP (2024). First validation of the childhood lupus low disease activity state (cLLDAS) definition in a real-life longitudinal cSLE cohort. Clin Immunol.

[25] Hochberg MC (1997). Updating the American College of Rheumatology revised criteria for the classification of systemic lupus erythematosus. Arthritis Rheum.

[26] Petri M, Orbai A-M, Alarcón GS (2012). Derivation and validation of the Systemic Lupus International Collaborating Clinics classification criteria for systemic lupus erythematosus. Arthritis Rheum.

[27] Aringer M, Costenbader K, Daikh D (2019). 2019 European League Against Rheumatism/American College of Rheumatology classification criteria for systemic lupus erythematosus. Ann Rheum Dis.

[28] Panyawong W, Pavasuthipaisit C, Santitadakul R (2020). Validation of the Thai Version of the Patient Health Questionnaire for Adolescents (PHQ-A) in adolescent psychiatric patients: Validation of the Thai version of the PHQ-A. Int J Child Dev Ment Health.

[29] Tiirikainen K, Haravuori H, Ranta K (2019). Psychometric properties of the 7-item Generalized Anxiety Disorder Scale (GAD-7) in a large representative sample of Finnish adolescents. Psychiatry Res.

[30] Buysse DJ, Reynolds CF, Monk TH (1989). The Pittsburgh sleep quality index: A new instrument for psychiatric practice and research. Psychiatry Res.

[31] Sitasuwan T, Bussaratid S, Ruttanaumpawan P (2014). Reliability and validity of the Thai version of the Pittsburgh Sleep Quality Index. J Med Assoc Thai.

[32] Varni JW, Burwinkle TM, Szer IS (2004). The PedsQL Multidimensional Fatigue Scale in pediatric rheumatology: reliability and validity. J Rheumatol.

[33] Broadbent E, Petrie KJ, Main J (2006). The brief illness perception questionnaire. J Psychosom Res.

[34] Sudjaritruk T, Aurpibul L, Songtaweesin WN (2021). Integration of mental health services into HIV healthcare facilities among Thai adolescents and young adults living with HIV. J Int AIDS Soc.

[35] Gladman DD, Ibañez D, Urowitz MB (2002). Systemic lupus erythematosus disease activity index 2000. J Rheumatol.

[36] Piga M, Chessa E, Morand EF (2022). Physician Global Assessment International Standardisation COnsensus in Systemic Lupus Erythematosus: the PISCOS study. Lancet Rheumatol.

[37] Jump RL, Robinson ME, Armstrong AE (2005). Fatigue in systemic lupus erythematosus: contributions of disease activity, pain, depression, and perceived social support. J Rheumatol.

[38] Kawka L, Schlencker A, Mertz P (2021). Fatigue in Systemic Lupus Erythematosus: An Update on Its Impact, Determinants and Therapeutic Management. J Clin Med.

[39] Wahadat MJ, van den Berg L, Timmermans D (2021). LLDAS is an attainable treat-to-target goal in childhood-onset SLE. Lupus Sci Med.

[40] Cleanthous S, Tyagi M, Isenberg DA (2012). What do we know about self-reported fatigue in systemic lupus erythematosus?. Lupus (Los Angel).

[41] Fanouriakis A, Kostopoulou M, Andersen J (2024). EULAR recommendations for the management of systemic lupus erythematosus: 2023 update. Ann Rheum Dis.

