# Supplementary material for: Mental health monitoring in adolescents with SLE: associations with lupus low disease activity state and remission
Source: Lupus Sci Med. 2026 Jun 17;13(1):e002034. doi: 10.1136/lupus-2026-002034 (PMC13289191; doi:10.1136/lupus-2026-002034)
Supplement: online supplemental table 1 [file lupus-13-1-s001.docx]

**Supplement table 1** Clinical and laboratory characteristics

| **Clinical and laboratory characteristics** | **1^st^ Visit**  **(n=117)** | **2^nd^ Visit**  **(n= 117)** | ***p*-value** | |
| --- | --- | --- | --- | --- |
| **SLE presentation** |  |  |  | |
| Mucocutaneous, n (%) | 11 (9.4) | 9 (7.7) | 0.774 | |
| Musculoskeletal, n (%) | 4 (3.4) | 2 (1.7) | 0.687 | |
| Hematologic, n (%) | 4 (3.4) | 4 (3.4) | 1.00 | |
| Active lupus nephritis, n (%) | 11 (9.4) | 12 (10.3) | 1.000 | |
| **Laboratory** |  |  |  | |
| Hemoglobin, g/L; mean ± SD | 11.8±1.6 | 11.9±1.6 | 0.451 | |
| White blood cell count, /mcL; mean ± SD | 6,692.4±2462.8 | 6,630.52±3,112.8 | 0.819 | |
| Platelet count, /mcL; mean ± SD | 300,153.9±79,787.8 | 307,179.5±100,641.1 | 0.398 | |
| Erythrocyte sedimentation rate, mm/hr; mean ± SD | 16.5 (8, 32.8) | 13 (7.8, 3) | 0.614 | |
| Serum creatinine, mg/dL; mean ± SD | 0.6±0.2 | 0.6±0.3 | **0.025** | |
| Urine protein per creatinine ratio, mg/mg; median (IQR) | 0.1 (0.1, 0.2) | 0.1 (0.1, 0.3) | 0.898 | |
| Complement C3 level, mg/dL; mean ± SD | 92.7±25.6  (n=114) | 90.8±26.4  (n=108) | | 0.376 |
| Complement C4 level, mg/dL; mean ± SD | 17.7±9.1  (n=74) | 18.2±9.2  (n=76) | | 0.437 |

^IQR, interquartile range; SD, standard deviation^

*^p^* ^< 0.05 is considered a significant level^

**Supplement table 2.** Intercorrelations among SLEDAI-2K, physician global assessment, depressive symptoms, anxiety, sleep disturbance, fatigue, illness perception, and pain

| SECOND VISIT | Prednisolone dose (mg/kg/day) | SLEDAI-2K score | PGA score | PHQ-A, score | GAD-7, score | PSQI, score | PedsQL-MFS, total score | PedsQL-General | PedsQL-Sleep/ Rest | PedsQL-Cognitive | B-IPQ, score |
| --- | --- | --- | --- | --- | --- | --- | --- | --- | --- | --- | --- |
| PGA score | **0.198**  **(p=0.032)** | **0.520**  **(p<0.001)** |  |  |  |  |  |  |  |  |  |
| PHQ-A, score | 0.011  (p=0.904) | 0.021  (p=0.615) | 0.059  (p=0.527) |  |  |  |  |  |  |  |  |
| GAD-7, score | -0.052  (p=0.579) | 0.035  (p=0.822) | 0.074  (p=0.430) | **0.634**  **(p<0.001)** |  |  |  |  |  |  |  |
| PSQI, score | 0.038  (p=0.678) | 0.021  (p=0.822) | 0.113  (p=0.225) | **0.601**  **(p<0.001)** | **0.459**  **(p<0.001)** |  |  |  |  |  |  |
| PedsQL-MFS, total score | 0.029  (p=0.760) | -0.076  (p=0.481) | -0.094  (p=0.312) | **-0.804**  **(p<0.001)** | **-0.573**  **(p<0.001)** | **-0.596**  **(p<0.001)** |  |  |  |  |  |
| PedsQL--General | 0.038  (0.683) | -0.048  (p=0.610) | -0.088  (p=0.348) | **-0.661**  **(p<0.001)** | **-0.483**  **(p<0.001)** | **-0.461**  **(p<0.001)** | **0.849**  **(p<0.001)** |  |  |  |  |
| PedsQL-Sleep/ Rest | -0.031  (p=0.737) | -0.130  (p=0.162) | -0.106  (p=0.253) | **-0.677**  **(p<0.001)** | **-0.481**  **(p<0.001)** | **-0.561**  **(p<0.001)** | **0.868**  **(p<0.001)** | **0.622**  **(p<0.001)** |  |  |  |
| PedsQL-Cognitive | 0.078  (p=0.404) | -0.028  (p=0.763) | -0.070  (p=0.454) | **-0.720**  **(p<0.001)** | **-0.552**  **(p<0.001)** | **-0.535**  **(p<0.001)** | **0.877**  **(p<0.001)** | **0.651**  **(p<0.001)** | **0.600**  **(p<0.001)** |  |  |
| B-IPQ, score | -0.045  (p=0.634) | 0.025  (p=0.787) | 0.144  (p=0.121) | **0.507**  **(p<0.001)** | **0.432**  **(p<0.001)** | **0.421**  **(p<0.001)** | **-0.622**  **(p<0.001)** | **-0.632**  **(p<0.001)** | **-0.467**  **(p<0.001)** | **-0.525**  **(p<0.001)** |  |
| VAS-P score | -0.033  (p=0.720) | -0.42  (p=0.651) | 0.057  (p=0.543) | **0.366**  **(p<0.001)** | **0.299**  **(p=0.001)** | **0.305**  **(p=0.001)** | **-0.406**  **(p<0.001)** | **-0.375**  **(p<0.001)** | **-0.300**  **(p=0.001)** | **-0.411**  **(p<0.001)** | **0.300**  **(p=0.001)** |

^B-IPQ, Brief Illness Perception Questionnaire; GAD-7, Generalized Anxiety Disorder-7; PedsQL-MSF, PedsQL Multidimension Fatigue Scale; PGA, physician global assessment; PHQ-A, Patient Health Questionnaire for Adolescents; PSQI, Pittsburgh Sleep Quality Index; SLEDAI-2K, SLE disease activity index 2000; VAS-P, visual analogue scale for pain^

^A correlation coefficient (r) between 0.6 and 1.0 (or -0.6 and-1.0) was considered a strong correlation.^

^r between 0.4 and 0.6 (or -0.4 and -0.6) was moderate correlation.^

^r less than 0.4 (or -0.4) was a weak correlation.^

*^p^* ^< 0.05 is considered a significant level^

**Supplement table 3** Comparison of disease state and mental health between adolescent SLE with and without a history of lupus nephritis at first and second visits

| Variable | **1^st^ Visit** | | p-value | **2^nd^ Visit** | | p-value |
| --- | --- | --- | --- | --- | --- | --- |
|  | History of LN  (n= 75) | No History of LN  (n= 42) |  | History of LN  (n = 75) | No History of LN  (n = 42) |  |
| cLLDAS, n (%) | 50 (66.7) | 26 (61.9) | 0.687 | 53 (70.4) | 32 (76.2) | 0.666 |
| cSLE Remission, n (%) | 20 (26.7) | 12 (28.6) | 0.494 | 23 (30.7) | 19 (45.2) | 0.085 |
| SLEDAI-2K, score; median (IQR) | 2 (0, 4) | 2 (0, 4) | 0.094 | 2 (0, 4) | 0 (0, 2) | **0.025** |
| PGA (scale 0-3), score; median (IQR) | 0 (0, 0.3) | 0 (0, 0.3) | 0.33 | 0 (0, 0.3) | 0 (0, 0) | **0.022** |
| Prednisolone doses, mg/day; median (IQR) | 5 (5, 10) | 5 (2.5, 10) | 0.507 | 5 (5, 5) | 5 (2.5, 5) | **0.021** |
| Prednisolone doses, mg/kg/day; median (IQR) | 0.11 (0.08, 0.18) | 0.10 (0.05, 0.17) | 0.226 | 0.11 (0.08, 0.14) | 0.08 (0.05, 0.13) | **0.019** |
| PHQ-A, score; median (IQR) | 3 (1, 6) | 4 (2, 5) | 0.203 | 2 (1, 5) | 3 (1, 5) | 0.288 |
| GAD-7, score; median (IQR) | 2 (0, 5) | 3 (1, 5) | 0.244 | 2 (1, 4) | 2 (0, 4) | 0.501 |
| PSQI, score; median (IQR) | 4 (2, 6.5) | 4 (3, 6) | 0.471 | 4 (2, 5.5) | 3 (2, 6) | 0.962 |
| PedsQL-MFS, total score; mean ± SD | 78.4±15.5 | 73.4±16.2 | 0.101 | 77.6±16.5 | 76.8±15.2 | 0.789 |
| General | 82.6±15.4 | 78.6±18.6 | 0.215 | 82.5±15.6 | 83.5±14.3 | 0.733 |
| Sleep/ Rest | 75.0±19.7 | 69.2±19.9 | 0.132 | 72.0±21.7 | 73.7±16.9 | 0.658 |
| Cognitive | 78.0±18.3 | 73.3±19.7 | 0.194 | 78.4±19.7 | 73.7±20.7 | 0.221 |
| B-IPQ, score, median (IQR) | 19 (10.5, 30) | 17 (12, 31) | 0.962 | 18 (11.5, 29.5) | 15 (7, 26) | 0.356 |
| VAS-P, score; median (IQR) | 0 (0, 2) | 0 (0, 1) | 0.899 | 0 (0, 2) | 0 (0, 2) | 0.213 |

^B-IPQ, Brief Illness Perception Questionnaire; cLLDAS, childhood lupus low disease activity state; cSLE, childhood-onset SLE; GAD-7, Generalized Anxiety Disorder-7; IQR, interquartile range; LN, lupus nephritis; PedsQL-MSF,^ ^PedsQL Multidimension Fatigue Scale; PGA, physician global assessment; PHQ-A, Patient Health Questionnaire for Adolescents; PSQI, Pittsburgh Sleep Quality Index; SD, standard deviation; SLEDAI, SLE disease activity index 2000; VAS-P, visual analogue scale for pain^

*^p^* ^< 0.05 is considered a significant level^

**Supplement table 4 Comparison of prednisolone doses in different disease activity states**

| **Prednisolone doses, mg/kg/day; median (IQR)** | **1^st^ Visit** | | p-value | **2^nd^ Visit** | | p-value |
| --- | --- | --- | --- | --- | --- | --- |
|  | Achieved cLLDAS  n=75 | Non-achieved cLLDAS  n=42 |  | Achieved cLLDAS  n=85 | Non-achieved cLLDAS  n=32 |  |
|  | 0.09 (0.06, 0.10) | 0.29 (0.15, 0.49) | **<0.001** | 0.08 (0.06, 0.11) | 0.24 (0.16, 0.49) | **<0.001** |
|  | cSLE-remission  n=32 | Non-remission  n=85 |  | cSLE-remission  n=42 | Non-remission  n=75 |  |
|  | 0.07 (0.05, 0.10) | 0.11 (0.08, 0.23) | **<0.001** | 0.08 (0.05, 0.10) | 0.12 (0.09, 0.30) | **<0.001** |
|  | Active SLE  (n=18) | Inactive SLE  (n=99) |  | Active SLE  (n=14) | Inactive SLE  (n=103) |  |
|  | 0.16 (0.09, 0.38) | 0.10 (0.07, 0.15) | **0.009** | 0.28 (0.10, 0.62) | 0.10 (0.07, 0.12) | **<0.001** |

^cLLDAS, childhood lupus low disease activity state; cSLE, childhood-onset SLE; IQR, interquartile range^

*^p^* ^< 0.05 is considered a significant level^
